# Validation of an 18-item version of the Swedish Knee Self-Efficacy Scale for patients after ACL injury and ACL reconstruction

**DOI:** 10.1186/s40634-021-00414-2

**Published:** 2021-10-25

**Authors:** S. Beischer, E. Hamrin Senorski, P. Thomeé, R. Thomeé

**Affiliations:** 1 Unit of Physiotherapy, Department of Health and Rehabilitation, Institute of Neuroscience and Physiology, The Sahlgrenska Academy, University of Gothenburg, Box 455, SE-405 30 Gothenburg, Sweden; 2 Sportrehab Sports Medicine Clinic, Stampgatan 14, SE-411 01 Gothenburg, Sweden; 3 Department of Orthopaedics, Sahlgrenska University Hospital, Mölndal, Sweden

**Keywords:** Anterior cruciate ligament, Cross-cultural adaptation, Patient-reported outcome measure, Psychometrics, Self-efficacy

## Abstract

**Purpose:**

To evaluate the measurement properties of a new version of the Swedish Knee Self-Efficacy Scale (K-SES) in samples of individuals with an anterior cruciate ligament (ACL) injury and after ACL reconstruction. A secondary aim was to translate the new version of K-SES into English in order to prepare for future complete cross-cultural adaptation.

**Methods:**

The reliability, structural validity, internal consistency and construct validity of the new, 18-item version of the K-SES (K-SES_18_) were assessed according to the COnsensus-based Standards for the selection of health Measurement INstruments (COSMIN) checklist for evaluating methodological quality. The Swedish version of the K-SES_18_ was translated to English using recommended guidelines.

**Results:**

The test-retest reliability for the K-SES_18_ subscale *present* and the K-SES_18_ subscale *future* showed an Interclass Correlation Coefficient (ICC) = 0.92. In addition, the K-SES_18_ had a Cronbach’s α ranging from 0.93 to 0.96 for the K-SES_18_ subscale *present* and from 0.81 to 0.91 for the K-SES_18_ subscale *future*. No floor and ceiling effects were identified for the subscale *present* or the subscale *future* of the K-SES_18_. A factor analysis produced 2 factors of importance; K-SES_18_*present* and K-SES_18_*future*. Seven predefined hypotheses were confirmed.

**Conclusion:**

The K-SES_18_ has acceptable reliability and validity to assess knee self-efficacy in patients up to 18 months after ACL injury and reconstruction.

**Level of evidence:**

IV.

**Supplementary Information:**

The online version contains supplementary material available at 10.1186/s40634-021-00414-2.

## Background

High self-efficacy is associated with a greater return to sports rate, along with other positive psychological responses, such as high motivation, high confidence, and low fear of re-injury [3, 31, 37]. A vast majority of patients who are treated with anterior cruciate ligament (ACL) reconstruction and rehabilitation expect to return to sport (RTS) within 12 months from surgery [13]. However, only about one in two patients return to competitive level of sports [2, 4]. The discrepancy between patients’ expectations and the proportion of patients that RTS can partly be explained by patients’ negative psychological responses, such as anxiety, depression and loss of athletic identity [15, 27].

Self-efficacy is defined as the belief in one’s ability to succeed or accomplish a task in order to achieve a specific outcome [6]. Furthermore, self-efficacy is influenced by one’s initiative for action, level of effort and resilience to setbacks, as well as previous experience of failure and success, including observation of oneself and others [6]. Higher levels of self-efficacy have repeatedly been associated with enhanced outcome in patients with diseases and impairments [9, 16, 21, 22, 28, 39]. With regard to knee joint disorders, a positive association between greater self-efficacy and positive outcomes has been reported for patients with knee osteoarthritis [9, 10, 21], total joint arthroplasty [40, 43], and meniscectomy [11]. To assess perceived knee-related self-efficacy in patients with ACL injury or reconstruction, the Knee Self-Efficacy Scale (K-SES) was developed in 2006 by Thomeé et al. [35] The original version of the K-SES has been used by different research groups in Sweden [1, 8, 14] and has been cross-culturally adapted into Dutch [41] and English [12]. The original K-SES is used to identify patients with high self-efficacy as well as patients with low self-efficacy from early after ACL injury and ACL reconstruction up to 12 months thereafter. A higher preoperative knee-related self-efficacy has been associated with better knee function in sport and recreational activities, quality of life, greater frequency of physical activity and acceptable hop performance 1 year after ACL reconstruction [37]. In clinic practice, identifying patients that report low scores is therefore important as these patients’ self-efficacy can be strengthened during rehabilitation using proper strategies [36].

The original version of the K-SES is a self-administrated questionnaire and consists of 2 subscales; *present knee self-efficacy (present)*, consisting of 18 items, and *future knee self-efficacy (future)*, consisting of 4 items. Patients rate each item on an 11-point Likert scale, ranging from 0 = *not at all certain* to 10 = *very certain* [37]. Each subscale is scored independently by calculating the mean score of item 1–18 (*present knee self-efficacy*) and item 19–22 (*future knee self-efficacy)*. For injured patients, who had not undergone an ACL-reconstruction, item 22 is excluded. Good validity has been reported for the original version of the K-SES for patients with an ACL injury and after ACL reconstruction aged 16 to 60 years [35]. Furthermore, acceptable test-retest reliability (interclass correlation coefficient [ICC] = 0.75) was shown for patients who had undergone ACL reconstruction, 2–3 months postoperatively [35].

The primary aim of this study was to evaluate the measurement properties of a new, 18-item version of the Swedish Knee Self-Efficacy Scale (K-SES) in samples of individuals with an anterior cruciate ligament (ACL) injury and after ACL reconstruction. A secondary aim was to translate the new version of K-SES into English in order to prepare for future complete cross-cultural adaptation.

## Method

After many years of experience using the K-SES, we have developed an 18-item version after having considered suggestions from patients with an ACL injury and from patients after ACL reconstruction, as well as suggestions from colleagues with many years of clinical experience. As many complaints arose during the years regarding four items (jumping ashore, running after children, horseback riding, moving around on a small boat, and working out hard shortly after an injury) in the subscale *present* of the original K-SES it was decided to remove these five items. In addition, there was an agreement on adding 1 item (cleaning at home). To reach a better understanding of 1 item of the K-SES subscale *present* and 3 items of the subscale *future* these items were rephrased. A pilot test of the modified version was conducted with 5 clinicians and 5 patients expressing their understanding of the new items. No changes were made after the pilot test. Thus, the new version consist of 18 items (K-SES_18_). The K-SES_18_ is, like the original version, a self-administrated questionnaire and scored in the same way. In patients who had not undergone an ACL reconstruction, item 18 of the K-SES_18_ “How certain are you that your knee will get better than before surgery” is excluded.

Table 1 presents detailed information about the modification of the original K-SES.

**Table 1:** The modification of the original version of the Knee Self-Efficacy Scale

**Original**	**Modification**	**18-item version**
**Present**		**Present**
***A - Daily activities***		***A - Daily activities***
*How certain are you about:*		*How certain are you about:*
1 taking a walk in the forest		1 walking in the forest
2 climbing up and down stairs		2 walking down stairs/down hill
3 going out dancing	*moved to section B*	3 running to catch the bus
4 jumping ashore	*deleted*	4 working in the garden
5 running after children	*deleted*	5 cleaning at home
6 running for the tram/bus	*rephrased*	
7 working in the garden		
***B - Sport and leisure activities***		***B - Sport and leisure activities***
*How certain are you about:*		*How certain are you about:*
1 bicycling long distance		1 cycling longer distance
2 cross-country skiing		2 cross-country skiing
3 horseback riding	*deleted*	3 swimming
4 swimming		4 dancing
5 hiking in the mountains		5 hiking in the mountains
***C - Physical activities***		***C - Physical activities***
*How certain are you about:*		*How certain are you about:*
1 squatting		1 squatting
2 jumping sideways from one leg to the other		2 jumping sideways from one leg to the other
3 working out hard a short time after an injury	*deleted*	3 hopping on the injured leg
4 performing a one-leg hop on the injured leg		4 quickly changing direction
5 moving around in a small boat	*deleted*	
6 doing fast twisting		
**Future**		**Future**
***D - Your knee function in the future***		***D - Your knee function in the future***
*How certain are you that:*		*How certain are you that:*
1 you can participate on the same activity levels before the injury	*rephrased*	1 your knee will get well
2 you will not have new knee injuries		2 you can return to the same physical activity level as before your injury
3 you that your knee will not “break down”	*rephrased*	3 you will not have new knee injuries
4 your knee will not get worse than before surgery (only if you have had surgery)	*rephrased*	4 your knee will get better than before surgery (only if you have undergone surgery)

### Evaluation of measurement properties of the 18-item version of the Knee Self-Efficacy Scale

The reliability, structural validity, internal consistency and construct validity of the K-SES_18_ were assessed according to the COnsensus-based Standards for the selection of health Measurement INstruments (COSMIN) checklist for evaluating methodological quality [24].

#### Patient samples

Two cohorts of patients with an ACL injury, who had or had not undergone an ACL reconstruction, were included in this study. Ethic approval has been obtained from the Regional Ethical Review Board in Gothenburg and the Swedish Ethical Review Authority (registration numbers: 265–13, T023–17, 2020–02501).

Cohort 1 was included for a test-retest analysis of the K-SES subscales *present* and *future,* respectively, and consisted of 32 patients (50% females, mean age 28.9 ± 10.3, min-max 16–50 years) who had undergone ACL reconstruction. The patients were recruited from a sports medicine clinic and had a median (Interquartile Range [IQR]) physical activity level, measured with a modified version of the Tegner Activity Scale (Tegner) [32], of 3 (2.8) ranging from Tegner level 1 to 7. A complete version of the modified version of Tegner cannot be found in the literature, and therefore a Swedish and an English version is presented in Additional file 1. The patients’ median (IQR) frequency and intensity of physical activity, measured with the Physical Activity Scale (PAS) [17, 18], was 2 (1.0) ranging from 1 to 4.

Cohort 2 was included for analyses of internal consistency and construct validity of the K-SES subscales *present* and *future* and consisted of 1865 patients from a rehabilitation registry, Project ACL, established in September 2014 and located in Gothenburg, Sweden. In February 2021, the registry comprised almost 3000 patients with an ACL injury. Patients included in the registry, are regularly evaluated with Patient Reported Outcomes (PROs) and a battery of tests consisting of muscle strength and hop tests. The evaluations follow a predetermined schedule of follow-ups at 10 weeks, 4, 8, 12 and 18 months, 2 and 5 years and then every 5th year after ACL injury or reconstruction. In addition, patients who undergo an ACL reconstruction are evaluated within 6 weeks before the ACL-reconstruction. Patients are continuously included into the registry regardless of how long time has passed since their ACL injury or reconstruction. Therefore, some patients only have data from the early evaluations, some only from the later evaluations, and, others from all evaluations. Data on patient demographics and results from PROs were extracted from the registry in May 2018. Eleven follow-ups were used in the analyses: 10 weeks, 4, 8, 12 and 18 months after ACL injury, within 6 weeks prior to ACL reconstruction and 10 weeks, 4, 8, 12 and 18 months after ACL reconstruction. Patient demographics for included patients at each follow-up are presented in Table 2.

**Table 2:** Patient demographics of cohort 2

**Follow-ups**	**n**	**Age**	**Female (%)**	**BMI**	**Tegner Pre-injury activity level**
10 weeks after ACL injury	228	32.2 ± 13.2 (12.3;63)	59	24 ± 3 (15;38)	7 (1;10) (4)
4 months after ACL injury	206	33.8 ± 13.1 (8.7;65.7)	59	24 ± 3 (15;39)	7 (1;10) (4)
8 months after ACL injury	167	36.0 ± 12.6 (9.1;66.0)	59	24 ± 3 (16;37)	6 (1;10) (4)
12 months after ACL injury	133	37.7 ± 12.3 (9.4;66.3)	61	25 ± 4 (15;37)	6 (1;10) (4)
18 months after ACL injury	88	38.2 ± 11.8 (9.9;66.8)	58	25 ± 4 (16;39)	6 (1;10) (3)
Preoperative^a^	388	27.9 ± 10.3 (13.4;60.9)	64	24 ± 3 (17;39)	8 (1;10) (3)
10 weeks after ACL reconstruction	913	27.5 ± 10.1 (11.5;64.0)	57	24 ± 3 (15;39)	8 (1;10) (3)
4 months after ACL reconstruction	929	27.7 ± 10.0 (11.6;64.1)	56	24 ± 3 (15;39)	8 (1;10) (3)
8 months after ACL reconstruction	877	28.5 ± 10.5 (12.6;64.5)	55	24 ± 3 (18;39)	8 (1;10) (3)
12 months after ACL reconstruction	715	28.7 ± 10.5 (13.0;64.8)	56	24 ± 3 (17;43)	8 (1;10) (3)
18 months after ACL reconstruction	438	30.3 ± 11.1 (13.5;65.3)	55	24 ± 3 (18;36)	8 (1;10) (3)

*ACL injury* refers to patients treated with rehabilitation, *ACL reconstruction* refers to patients treated with reconstruction and rehabilitation, *Age* and *Body Mass Index (BMI)* is presented as mean ± standard deviation (min;max), *Tegner Pre-injury activity level* is presented as median (min;max) and (interquartile range)

^a^Follow-up performed within 6 weeks prior to ACL reconstruction

### Reliability

To determine test-retest reliability of the K-SES_18_ subscales *present* and *future,* respectively, the patients completed the K-SES_18_ with a minimum of 10 days between test and retest. To be included in the test–retest evaluation, the patients’ condition was regarded as clinically stable during the 10-day period, and, 10 days were deemed sufficient to minimize recall bias. All patients had undergone ACL reconstruction and completed the test-retest between 4 and 12 months after reconstruction. The test-retest reliability was considered good if the ICC was higher than 0.70 [34].

Internal consistency is the degree of interrelatedness between items [25] and it was deemed good if Cronbach’s alpha was between 0.70 and 0.95 for the subscales [33].

### Floor and ceiling effects

Floor and ceiling effects, as a reflection of content validity of the total score of each subscale of the K-SES_18_ and each item, respectively, were considered to be present if more than 15% of the participants achieved the lowest (0) or highest score (10) of the mean score of each subscale and each item individually [33].

### Construct validity

Construct validity includes structural validity, hypotheses testing and cross-cultural adaptation, and is defined as the degree to which the scores of a PRO are consistent with à priori hypotheses, based on the assumption that the instrument validly measures the construct to be measured [25]. To determine construct validity, results from the PROs: the Knee injury and Osteoarthritis Outcome Score (KOOS), the ACL Return to Sport after Injury (ACL-RSI), the Tegner [32], the Physical Activity Scale (PAS) [17, 35], and each subscale of the K-SES_18_ were extracted from the rehabilitation registry. In the registry, all questionnaires are self-administrated and digitally distributed to the patients at the pre-defined follow-ups.

The KOOS [29] is used to assess patients’ opinions of their knee and associated problems and comprises 42 items in 5 subscales; pain (9 items), other symptoms (7 items), activities of daily living (17 items), function in sport and recreation (5 items), and knee-related quality of life (4 items). Each subscale score is calculated independently, by dividing the mean score of the individual items of each subscale and divided by 4 and then multiplying the result by 100 (100 indicates no problem and 0 indicates extreme problems). The KOOS has been reported to have acceptable test-retest reliability, with an ICC ranging from 0.85 to 0.93 for each subscale for patients with an ACL injury or after an ACL reconstruction [29]. In the rehabilitation registry, the subscale of activities of daily living is excluded except from the follow-ups at 6 weeks within the ACL-reconstruction and at 1, 2 and 5 years after ACL injury/reconstruction.

The ACL-RSI scale [20, 42] is used to assess psychological readiness to return to sports participation after ACL injury. The ACL-RSI measures 3 types of response with 12 items associated with the resumption of sport after an injury: emotions (5 items), confidence in performance (5 items) and risk appraisal (2 items). Patients are asked to rate each item on a 10-point Likert scale that range from 1 = the worse score and 10 = the best [20]. Higher scores reflect a positive psychological response. The Swedish version of the ACL-RSI scale has been validated, and is considered internally consistent and reliable (ICC = 0.89) for patients aged 18–45 years after an ACL reconstruction [20].

The Tegner [32] is used to assess preinjury, present and future levels of physical activity and is graded from 1 to 10, with 1 representing the least strenuous knee activity and 10 representing the most strenuous knee activity, such as rugby and international soccer. The Tegner has been reported to have acceptable test-retest reliability with an ICC of 0.8 for patients with ACL injury and for patients after ACL reconstruction [38]. In the present study, a new version of the Tegner was used (Additional file 1). The new version contains updated and more physical activities on each of the 10 levels. In the new version the “0” value, which represents “sick leave or disability pension because of knee problems” is omitted. Furthermore, the original version of the Tegner includes recreational sports as a choice up to level 7 and the modified Tegner up to level 9.

The PAS is used to assess patients’ frequency and intensity of physical activity. The PAS originates from a validated score for middle-aged and former athletes [30] and was modified by an expert group consisting of experienced physiotherapists and orthopedic surgeons, which assured good face validity for the scale [38]. The PAS is graded from 1 to 4 and the patient is asked to make their own assessment of how frequent and intense they participate in physical activity at the present time and prior to their ACL injury.

#### Structural validity

To assess structural validity of each of the K-SES subscales *present* and *future*, a maximum likelihood factor analysis using Harris Kaiser’s rotation method, as was used for the original version of K-SES [35], was applied to the K-SES_18_. As the original version of the K-SES includes two factors, it was hypothesized that the K-SES_18_ would include two factors as well, with items 1–14 loading on the first factor (*present* K-SES_18_) and items 15–18 loading on the second factor (*future* K-SES_18_).

#### Hypothesis testing

Hypothesis testing was performed using Spearman’s correlation coefficient for non-parametric data, to compare each subscale of the K-SES_18_ with the subscales of the KOOS and the ACL-RSI. The following cut-offs was used for interpretation of the correlation coefficient: 0.90–1.00 *very high correlation,* 0.70–0.90 *high correlation;* 0.50–0.70 *moderate correlation;* 0.30–0.50 *low correlation* < 0.30 *negligible correlation* [19]. The Mann–Whitney *U* test was used to examine differences in K-SES scores between various sub-groups (RTS versus not RTS; ACL injury/reconstruction ≤4 months versus ACL injury/reconstruction ≥8 months).

The following predefined hypotheses were developed:

1. Patients, who had been involved in knee-strenuous sports (defined as ≥ level 6 of the Tegner) and had returned to at least Tegner 6, would score higher on the subscale of present self-efficacy of the K-SES_18_ than patients who not have returned to at least Tegner 6, 8–18 months after ACL injury or reconstruction.
  *Reasoning*: Patients who RTS report a stronger psychological profile compared with patients with lower levels of physical activity [3, 31, 37].
2. Patients, who had returned to their previous level of sport, as measured with the Tegner, would score higher on the subscale of present self-efficacy of the K-SES_18_ than patients who had not returned to their previous level of sport, 8–18 months after ACL injury or reconstruction.
  *Reasoning*: Patients who RTS report a stronger psychological profile compared with patients with lower levels of physical activity [3, 31, 37].
3. Patients, who had sustained the ACL injury in the last 4 months, would score lower than the patients who have sustained the ACL injury ≥12 months ago.
  *Reasoning*: The perceived self-efficacy increase, at group level, during the course of the rehabilitation [38].
4. Patients, who had undergone ACL reconstruction in the last 4 months, would score lower than patients who had undergone an ACL reconstruction ≥12 months.
  *Reasoning*: The perceived self-efficacy increase, at group level, during the course of the rehabilitation [38].
5. The subscale pain, other symptoms, function in sports and recreation, and quality of life of the KOOS would correlate at least moderately (r_s_ ≥ 0.30) with the subscale of present self-efficacy, 8–18 months after injury and ACL reconstruction.
  *Reasoning*: Moderate to high correlations between the subscales of KOOS and the original version of the K-SES have been found at 12 months after injury and reconstruction [38].
6. The ACL-RSI would correlate at least moderately (r_s_ ≥ 0.30) with the subscale of present self-efficacy of the K-SES_18_, 8–18 months after ACL injury and reconstruction.
  *Reasoning*: The K-SES and the ACL-RSI was developed for similar patient groups and measure similar constructs and have previously been shown to correlate to each other [12, 20].
7. The ACL-RSI would correlate at least moderately (r_s_ ≥ 0.30) with the subscale of present and future self-efficacy of the K-SES_18__._
  *Reasoning*: The K-SES and the ACL-RSI was developed for similar patient groups and measure similar constructs and have previously been shown to correlate to each other [12, 20].

#### Translation

**Fig. 1:**
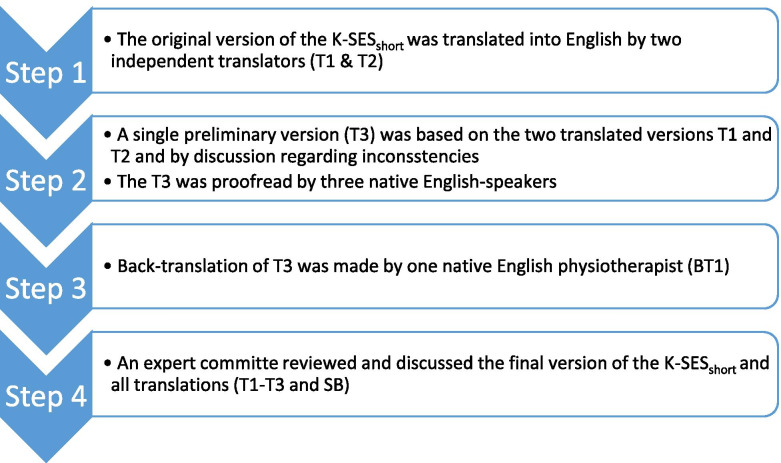
Illustration of the translation and cross-cultural adaptation process of the 18-item version of the Knee Self-Efficacy Scale (K-SES_18_)

The Swedish version of the K-SES_18_ was translated to English using recommended guidelines [7] (Fig. 1). For face validity, content validity and clarity, the translations were reviewed by an expert committee (ES/RT/SB), all physiotherapists and researchers with many years of experience of patients with ACL injury as well as of construction and validation of PROs. All discussion and changes during the translation processes were documented.

##### Step 1

The original version of the K-SES_18_ was translated from Swedish into English by 2 independent translators (T1 and T2) whereof both are native to the Swedish language and fluent in English. One translator (T2) was also native to the English language. One of the translators (T1) was one of the developers of the original K-SES. Both translators were aware of the concepts being measured and had previous experience in the use of the K-SES as researchers and physiotherapists.

##### Step 2

Minor inconsistencies between the 2 translated versions (T1 and T2) were resolved by discussion resulting in a single preliminary English version (T3). Two native English non-professionals and one native English physiotherapist, who was uninformed about the constructs being measured, proofread the preliminary English version (T3).

##### Step 3

The back-translation process of the preliminary version of the K-SES_18_ (T3) was performed by one native English physiotherapist (BT1).

##### Step 4

In order to combine all the translated versions into a preliminary version the two translators (ES/RT) and the co-author (SB) formed an expert committee. The committee reviewed and discussed the original version of the K-SES_18_ and all translations (T1, T2, T3, BT1) together with the corresponding written reports until a consensus was reached and an English version of the K-SES_18_ and a written report of the synthesis process were completed.

## Results

### Reliability

The test-retest reliability for the K-SES_18_ subscale *present* and the K-SES_18_ subscale *future*, based on cohort 1, had for both subscales an ICC = 0.92. In addition, Cronbach’s α, based on cohort 2, across the 11 different follow-ups ranged from 0.93 to 0.96 for the subscale *present* and from 0.81 to 0.91 for the subscale *future* (Table 3).

**Table 3:** Cronbach’s α at 11 follow-ups for patients with an ACL injury and after ACL reconstruction

Follow-ups	n	Cronbach’s α	
		K-SES_18_	K-SES_18_
		present	future
10 weeks after ACL injury	228	0.96	0.85
4 months after ACL injury	206	0.95	0.85
8 months after ACL injury	167	0.95	0.88
12 months after ACL injury	133	0.96	0.87
18 months after ACL injury	88	0.95	0.91
Preoperative^a^	388	0.95	0.82
10 weeks after ACL reconstruction	913	0.93	0.82
4 months after ACL reconstruction	929	0.93	0.81
8 months after ACL reconstruction	877	0.94	0.81
12 months after ACL reconstruction	715	0.95	0.83
18 months after ACL reconstruction	438	0.95	0.84

*ACL injury* refers to patients treated with rehabilitation, *ACL reconstruction* refers to patients treated with reconstruction and rehabilitation, *K-SES*_*18*_ the 18-item version of the Knee Self-Efficacy Scale

^a^Follow-up performed within 6 weeks before ACL reconstruction

### Floor and ceiling effects

Based on cohort 2, no floor and ceiling effects were identified, using the definition that floor and ceiling effects were present if more than 15% of the participants achieved the lowest (0) or highest score (10) of the mean score for the subscale *present* or the subscale *future* of the K-SES_18_ (Additional file 2). Floor and ceiling effects were seen for several items at some of the follow-ups. For example, items 1, 4 and 5 had ceiling effects, especially late, > 8 months, after injury or reconstruction. Moreover, floor effects were seen early after injury or reconstruction e.g. for items 7 and 14.

### Construct validity

To analyse construct validity, results from all follow-ups of cohort 2 were used, from 10 weeks after injury to 18 months after ACL reconstruction. The factor analysis with an eigenvalue set at > 1 produced 2 factors of importance; factor 1 was related to how the patients perceived their present physical performance and function, i.e. K-SES_18_*present*, while factor 2 was related to how the patients perceived their future physical performance and prognosis for their knee, i.e. K-SES_18_*future*.

### Hypothesis testing

All seven predefined hypotheses were confirmed for cohort 2, Tables 4, 5 and 6. Patients who previously had been involved in knee-strenuous sports and had returned to at least Tegner 6 scored higher on the subscale present self-efficacy of the K-SES_18_ than patients who had not returned to at least Tegner 6, 8–18 months after ACL injury, [8 months: median (min-max) 8.4 (5.6–10) vs. 7.2 (2.0–9.4); 12 months: 9.1 (6.4–10) vs. 7.1 (0.4–9.9); 18 months: 9.6 (5.5–10.0) vs. 7.9 (1.7–9.6)]. For patients who had undergone ACL reconstruction, the similar pattern was observed [8 months: 8.7 (3.4–10.0) vs. 7.6 (0.0–9.9); 12 months: 9.1 (4.2–10.0) vs. 7.7 (0.1–9.9); 18 months: 9.4 (5.8–10.0) vs. 7.8 (2.0–10.0)].

**Table 4:** Comparative scores (median and min-max) of the subscales present and future of the K-SES_18_ according to follow-up and return to sport

**Follow-ups**	**n**	**Present**	**Future**	**Follow-ups**	**n**	**Present**	**Future**
				Preoperative*	388	5.2 (0.1–10)	7.5 (0–10)
10 weeks after ACL injury	228	4.5 (0–9.7)	6.7 (0–10)	10 weeks after ACL reconstruction	913	4.0 (0.1–9.5)	7.5 (0–10)
4 months after ACL injury	206	5.2 (0.1–9.6)^a^	6.3 (0–10)	4 months after ACL reconstruction	929	5.9 (0.2–9.9)^a^	7.5 (0–10)
8 months after ACL injury	167	6.9 (0.3–10)	6.3 (0–10)	8 months after ACL reconstruction	877	7.9 (0–10)	7.5 (0–10)
RTS	42	6.7 (0.8–10)^b^	7.0 (1–9.6)	RTS	172	8.3 (0.4–10)^b^	8.0 (1.3–10)
Not RTS	125	6.3 (0–10)	6.9 (0.3–10)	No RTS	705	7.8 (0–10)	7.5 (0–10)
12 months after ACL injury	133	7.4 (0.4–10)	6.5 (0–10)	12 months after ACL reconstruction	715	8.5 (0.1–10)	7.5 (0–10)
RTS	48	7.7 (3.4–9.9)^b^	7.2 (2.5–10)	RTS	256	9.1 (0.7–10)^b^	8.3 (0–10)
Not RTS	85	7.1 (0.4–10)	6.3 (0–10)	No RTS	459	8.1 (0.1–10)	7.0 (0–10)
18 months after ACL injury	88	8.0 (1.7–10)	5.8 (0–10)	18 months after ACL reconstruction	438	8.7 (0.7–10)	7.5 (0–10)
RTS	31	7.7 (4.4–10)^b^	5.8 (1–10)	RTS	195	9.2 (0.7–10)^b^	8 (0.5–10)
Not RTS	57	8.0 (1.7–9.9)	6.0 (0–10)	No RTS	243	8.2 (2–10)	6.8 (0–10)

*ACL injury* refers to patients treated with rehabilitation, *ACL reconstruction* refers to patients treated with reconstruction and rehabilitation, *K-SES*_*18*_ the 18-item version of the Knee Self-Efficacy Scale, *RTS* returned to sport (defined as present level of physical activity as measured with the Tegner activity scale being ≥ to the level the patient had before the injury)

*follow-up performed within 6 weeks before ACL reconstruction, ^a^*p* < 0,001, 4 months vs 12 months, ^b^*p* < 0,001, returned to sport vs not returned to sport

**Table 5:** Correlation between the subscale present of the *K-SES*_*18*_, the KOOS subscales and the ACL-RSI

Follow-ups	KOOS	KOOS	KOOS	KOOS	KOOS	ACL-RSI
	pain	symptoms	ADL	sports	QoL	
	*r*	*r*	*r*	*r*	*r*	*r*
10 weeks after ACL injury	.627**	.548**		.758**	.632**	
4 months after ACL injury	.595**	.473**		.759**	.673**	
8 months after ACL injury	.666**	.521**		.755**	.741**	.481**
12 months after ACL injury	.628**	.569**	.742**	.814**	.775**	.594**
18 months after ACL injury	.758**	.680**		.760**	.718**	.639**
Preoperative^a^	.667**	.609**	.685**	.729**	.653**	
10 weeks after ACL reconstruction	.474**	.434**		.634**	.517**	
4 months after ACL reconstruction	.476**	.413**		.701**	.531**	
8 months after ACL reconstruction	.547**	.507**		.738**	.612**	.525**
12 months after ACL reconstruction	.592**	.515**	.630**	.746**	.657**	.608**
18 months after ACL reconstruction	.617**	.523**		.801**	.681**	.647**

*ACL* Anterior Cruciate Ligament, *ACL injury* refers to patients treated with rehabilitation, *ACL reconstruction* refers to patients treated with reconstruction and rehabilitation, *ACL-RSI* Anterior Cruciate Ligament Return to Sport Index, *KOOS* Knee injury and Osteoarthritis Outcome Score, *K-SES*_*18*_ the 18-item version of the Knee-Self-Efficacy Scale, *QoL* quality of life

**p < 0.01

**Table 6:** Correlation between the subscale future of the *K-SES*_*18*_ and the ACL-RSI

Follow-ups	*r*
8 months after ACL injury	0.644**
12 months after ACL injury	0.768**
18 months after ACL injury	0.739**
8 months after ACL reconstruction	0.664**
12 months after ACL reconstruction	0.759**
18 months after ACL reconstruction	0.774**

*ACL* Anterior Cruciate Ligament, *ACL injury* refers to patients treated with rehabilitation, *ACL reconstruction* refers to patients treated with reconstruction and rehabilitation, *ACL-RSI* Anterior Cruciate Ligament Return to Sport Index, *K-SES*_*18*_ the 18-item version of the Knee-Self-Efficacy Scale

**p < 0.01

#### Translation

The majority of the items in the Swedish version of the K-SES_18_ were translated into English without any disagreements. The two translators (EHS and RT) met via a video conference and the T3 was created after a consensus discussion. The primary differences between the T1 and T2 were wording, for example item 13; “Quickly changing direction” versus “performing quick turns”.

One of the three proofreaders identified some issues regarding the wording of the last item, item 18. After discussion in the expert committee the wording was changed from ‘*that your knee will get better’,* to ‘*that your knee will be better’*. No other changes were made in the T3.

Two of the proofreaders who reviewed T3 had opinions about the word *certain*. After discussion in the expert committee, *certain* was not replaced by the word *confident* as suggested by the proofreaders. *Confidence* is often seen in the literature as synonymous with *self-efficacy* but according to Albert Bandura [5] the construct of self-efficacy differs from the colloquial term ‘confidence’. Confidence is a nonspecific term that refers to strength of belief but does not necessarily specify what the certainty is about.

## Discussion

The Swedish version of K-SES_18_ had acceptable reliability and good validity to assess knee self-efficacy in patients, up to 18 months after both ACL injury and reconstruction. In addition, the K-SES_18_ was translated into English. All our analyses of the measurement properties of the K-SES_18_, except for the test-retest analysis (*n* = 32), were on cohort 2 based on large populations of patients, ranging from early after ACL injury/reconstruction to 18 months after ACL injury/reconstruction.

The test-retest analyses resulted in an ICC of 0.92 for the K-SES_18_ subscale *present* as well as for the K-SES_18_ subscale *future* and is considered good [34]. These results are in accordance with the results of the original version of the K-SES [35]. However, the test-retest reliability analysis was performed on patients who had undergone an ACL reconstruction. It is thus unknown if the good test-retest reliability is applicable for patients who had not undergone an ACL reconstruction. In addition, test-retest reliability was assessed after a minimum of 10 days, which deemed to minimise recall bias to an acceptable level. The COSMIN [24] recommends an interval of 14 days to minimize recall bias and, thus, it cannot be excluded that some recall of some items can be present in this study.

The internal consistency, e.g. the degree of interrelatedness between items [24], is deemed good if Cronbach’s alpha is between 0.70 and 0.95 [33]. When the alpha is too high (> 0.95) it may reflect unnecessary duplication of content across items. Therefore, the values between 0.93 and 0.96 for the subscale *present* of the K-SES_18_ may indicate that some of the 14 items could be removed without losing information. Especially could an item reduction be made possible if specific K-SES versions are constructed for sub-groups of patients after ACL injury or after ACL reconstruction.

Patient responses, for the 11 follow-ups, were spread across response options from 0 to 10 in the K-SES_18_ with no floor or ceiling effects for the total scores at any of the follow-ups. This is when strictly applying the definition of floor or ceiling effect, i.e. to be present if more than 15% of the participants achieved the lowest (0) or highest score (10). This is one reason why all follow-ups are presented for the reader in Additional file 2, where it can be seen that for example at 12 and 18 months after ACL reconstruction a ceiling effect seems present, even though no patients actually achieved the *highest* score. However, floor- and ceiling effects were seen for several independent items. The K-SES_18_ can, for different ages and physical activity levels, identify patients with a high self-efficacy, as well as patients with a low self-efficacy, from early after ACL injury or reconstruction up to 18 months thereafter. As rehabilitation progresses, a gradually higher self-efficacy was noted, which is what clinicians should aim for, as a high or strong self-efficacy is associated with better outcome [8, 26, 38]. However, from a clinical standpoint, identifying patients that report low scores are important. These patients can be helped by strengthening their self-efficacy during rehabilitation using proper strategies [36]. Even though floor and ceiling effects may indicate a lack of content validity [23, 24], it is our belief that ceiling effects are not as important when it comes to self-efficacy beliefs as measured with the K-SES_18_. In clinic practice, it might be more important to identify patients with low self-efficacy. In addition, K-SES_18_ is not a measure of the patients’ actual capability to execute the certain tasks described in each item, rather, it is how they *experience* their ability to execute the tasks [6].

The factor analysis of the K-SES_18_ produced the same two factors of importance as the original version of K-SES, i.e. the subscale *present* knee self-efficacy, consisting of 14 items, and the subscale *future* knee self-efficacy, consisting of 4 items. The original K-SES has previously been translated and culturally adapted into Dutch [41] and English [12]. The finding of two factors of importance, in accordance with the original version of the K-SES, was confirmed in the Dutch K-SES when using an explanatory factor analysis. However, in a confirmatory factor analysis, the two-factor model was not confirmed in neither the Dutch [41] nor the English version [12].

All seven hypotheses regarding the relationships between scores on the K-SES_18_ and the KOOS and the ACL-RSI were confirmed. Likewise, how the scores vary between different patients sub-groups, e.g. between patients who had and had not returned to sport, were also confirmed. The correlations between the K-SES_18_ and the KOOS and the ACL-RSI, respectively, varied between 0.43 and 0.81. At 8–18 months after ACL injury and reconstruction all correlations were considered strong except for the correlation between ACL-RSI and the K-SES_18_ (*r* = 0.48, *p* < 0.01) at the 8-month follow-up. There were moderate correlations 4 months after ACL injury, and 10 weeks and 4 months after ACL-reconstruction, which can be explained by the heterogeneity in symptoms and function among patients early after ACL reconstruction. In summary, it can be expected that K-SES_18_ has good construct validity as the explanatory factor analysis confirmed the two-factor model and as all of the pre-defined hypotheses were confirmed, even though results from a confirmatory factor analysis is unknown.

Thomeé et al. [38] reported a significant increase in the patients’ perceived self-efficacy during the rehabilitation processes after ACL injury and after ACL reconstruction. This pattern of increase in present self-efficacy was confirmed in the present study. However, in patients with an ACL injury and in patients with ACL reconstruction, there was no change in the median future self-efficacy from 10 weeks to 18 months after injury and reconstruction. The divergent results in the present study and the study by Thomeé et al. [38] might be explained by the large differences in sample sizes. In the present study, we included 88 to 929 patients, with a total of 1865 unique patients, at the 11 different follow-ups, compared to 104 patients in the original study [38]. This finding of no or only minor change of the perceived future self-efficacy at group level, might suggest that there is less need to assess this subscale repeatedly during the rehabilitation. However, it may still be useful for the clinician to evaluate perceived future self-efficacy on an individual level.

A limitation to the present study is that no formal co-design method to assess content validity of the K-SES_18_ was carried out. Therefore, important feedback from patients and clinicians might have been lost. However, a pilot test of the modified version was conducted with 5 clinicians and 5 patients expressing their understanding of the new items. No changes were made after the pilot test. Furthermore, the original version of the K-SES was developed thoroughly with a standardized process where both patients and different professional health care clinicians attended in the development process [35]. Future studies should pre-test the K-SES_18_ and in a standardised way, e.g. by focus-groups, gather feedback of the questionnaire. Another limitation of the present study is that the minimum and maximum of patients’ age varied across the different follow-ups. In addition, the median of Tegner activity level of 6 to 8 at the different follow-ups indicate that we included a relative active population. The generalisability of the present study might therefore be limited to active patients, 16–50 years of age, as for the original version of the K-SES. In the future, there might be a need to construct specific K-SES for various groups, such as for example K-SES_young_ (10–15 years of age), K-SES_elite athletes_ and K-SES_recreational athletes_ etc. Furthermore, no analysis regarding responsiveness was performed. Responsiveness is defined as the ability of an instrument to detect change over time in the construct being measured [25]. However, the confirmation of the third and the fourth hypothesis in the assessment of construct validity indicates that a change in self-efficacy through the course of the rehabilitation is captured. Patients who had sustained the ACL injury or had undergone ACL reconstruction in the last 4 months scored significant lower than patients who have sustained the ACL injury or had undergone an ACL reconstruction ≥12 months ago. However, further assessment of responsiveness of the K-SES_18_, using recommended methods, is needed to confirm theses expectations. Finally, we performed a translation of the K-SES_18_ into English. Yet, before using the translated version of the K- SES_18_ the questionnaire should be assessed for cultural adaptation and pre-tested in an English-speaking population of patients according to Beaton et al. [7].

## Conclusion

The Swedish version of the K-SES_18_ has acceptable reliability and validity to assess knee self-efficacy in patients up to 18 months after ACL injury and reconstruction.

## Supplementary Information

**Additional file 1.** Swedish and English versions of the Tegner Activity Scale (Tegner). A complete version of the modified version of Tegner in Swedish and English used in the study.

**Additional file 2. **The frequency of total score for the K-SES_18_ subscale *present* and the K-SES_18_ subscale *future* across follow-ups 10 weeks to 18 months after ACL-injury and ACL-reconstruction. Frequency diagrams of K-SES_18_ subscales *present* and *future* for all 11 follow-ups as an illustration of floor and ceiling effects.

## Data Availability

The dataset used and/or analyzed are available from the corresponding author on request.

## Abbreviations

ACL: Anterior cruciate ligament
ACL-RSI: ACL Return to Sport after Injury
COSMIN: COnsensus-based Standards for the selection of health Measurement INstruments
Future: The subscale *future knee self-efficacy*
ICC: Interclass correlation coefficient
IQR: Interquartile Range
KOOS: Knee injury and Osteoarthritis Outcome Score
K-SES: Knee Self-Efficacy Scale
K-SES_18_: 18-item version of the Knee Self-Efficacy Scale
PAS: Physical Activity Scale
Present: The subscale *present knee self-efficacy*
PROs: Patient Reported Outcomes
RTS: Return to sport
Tegner: Tegner Activity Scale
