# Supplementary material for: Validation of an 18-item version of the Swedish Knee Self-Efficacy Scale for patients after ACL injury and ACL reconstruction
Source: J Exp Orthop. 2021 Oct 25;8:96. doi: 10.1186/s40634-021-00414-2 (PMC8542523; doi:10.1186/s40634-021-00414-2)
Supplement: Supplementary file 1 — Additional file 1. Swedish and English versions of the Tegner Activity Scale (Tegner). A complete version of the modified version of Tegner in Swedish and English used in the study. [file 40634_2021_414_MOESM1_ESM.pdf]

Additional file 1

Tegner Swedish version		Aktiviteter
10	Aktiv idrott	Akrobatik, Am fotboll, Brottning, Fotboll på högsta elitnivå, Konståkning, Rugby
9	Motionsidrott	Akrobatik, Am fotboll, Brottning, Konståkning, Rugby
9	Aktiv idrott	Fotboll, Ishockey, Puckelpist
8	Motionsidrott	Puckelpist
8	Aktiv idrott	Backhoppning, Basketboll, Budo, Handboll, Innebandy, Längdhopp, Squash, Trestegshopp
7	Motionsidrott	Backhoppning, Basketboll, Budo, Fotboll, Handboll, Innebandy, Ishockey, Längdhopp, Squash, Tresteg
7	Aktiv idrott	Badminton, Höjdhopp, Stavhopp, Tennis, Utförsåkning, Volleyball
6	Motionsidrott	Badminton, Höjdhopp, Ishockey, Stavhopp, Tennis, Utförsåkning, Volleyball
6	Aktiv idrott	Bandy, Baseball, Hinderlöpning, Häcklöpning, Orientering, Telemark skiing, Snowboard
5	Arbete	Tung verkstad, Brandman, Militär, Skogsarbetare
5	Motionsidrott	Bandy, Baseball, Orientering, Telemark, Snowboard
5	Aktiv idrott	Boxning, Brännboll, Diskus, Fallskärmshopp, Fäktning, Gymping, Kulstötning, Längdskidor, Motorcross, Motionsspårlöpning, Slägga, Speedway, Spjut, Tyngdlyftning, Vattenskidor
4	Arbete	Bonde, Polis, Hamnarbetare, Plåtslagare, Murare, Byggarbetare
4	Motionsidrott	Boxning, Brännboll, Diskus, Fäktning, Gymping, Kulstötning, Längdskidor, Motorcross, Motionsspårlöpning, Slägga, Speedway, Spjut, Tyngdlyftning, Vattenskidor
4	Aktiv idrott	Bordtennis, Dans, Löpning på plant underlag, Vattenpolo, Windsurfing
3	Arbete	Jägmästare, Lätt verkstadsarbete, Lastbilschaufför, Målare, Trädgårdsarbetare, Renhållningsarbetare, Skådespelare, Snickare, Sotare, Städerska, Vägarbetare
3	Skogspromenader	
3	Motionsidrott	Bordtennis, Dans, Löpning på plant underlag, Vattenpolo, Windsurfing
3	Aktiv idrott	Bodybuilding, Bowling, Curling, Cykel, Golf , Segling, Simning, Ridning
2	Arbete	Affärsbiträde, Bilreparatör, Biträde, Förskollärare, Militär, Nattvakt, Servitör, Sjuksköterska
2	Gång på ojämt underlag	
2	Motionsidrott	Bodybuilding, Bowling, Curling, Cykel, Golf, Segling, Simning, Ridning
1	Arbete	Busschaufför, Flygare, Frisör, Kock, Kontor, Läkare, Lärare, Sekreterare
1	Gång på plant underlag	
1	Aktiv idrott	Bridge, Bågskytte, Kanot, Schack, Skytte

Aktiv idrott Regelbunden tävling och träning

Motionsidrott Idrott "bara för skojs skull"

Tegner English version		Activities
10	Sports	Gymnastics, American football, Wrestling, Competitive level football/soccer, Figure skating, Rugby
9	Recr. Sports	Gymnastics, American football, Wrestling, Figure skating, Rugby
9	Sports	Fotball/soccer, Ice hockey, Mogul skiing
8	Recr. Sports	Mogul skiing
8	Sports	Ski jumping, Basketball, Martial arts, Team handball, Floorball, Long jump, Squash, Triple jump
7	Recr. Sports	Ski jumping, Basketball, Martial arts, Football/soccer, Team handball, Floorball, Long jump, Squash, Triple jump
7	Sports	Badminton, High jump, Pole vault, Tennis, Alpine skiing, Volleyball
6	Recr. Sports	Badminton, High jump, Ice hockey, Pole vault, Tennis, Alpine skiing, Volleyball
6	Sports	Bandy, Baseball, Steeple chase, Hurdling, Orienteering, Telemark skiing, Snowboard
5	Work	Heavy labor/construction, Fire-fighter, Military, Forest worker
5	Recr. Sports	Bandy, Baseball, Orienteering, Telemark skiing, Snowboard
5	Sports	Boxing, Rounders, Discus, Skydiving, Fencing, Aerobics, Shot put, Cross-country skiing, Motocross, Running, Hammer throw, Speedway, Javelin throw, Weightlifting, Water skating
4	Work	Farmer, Police, Dock worker, Metal worker, Bricklayer, Construction worker
4	Recr. Sports	Boxing, Rounders, Discus, Fencing, Aerobics, Shot put, Cross-country skiing, Motocross, Running, Hammer throw, Speedway, Javelin throw, Weightlifting, Water skating
4	Sports	Table tennis, Dancing, Running on even surface, Water polo, Windsurfing
3	Work	Forester, Light labor work, Truck driver, Painter, Gardener, Cleaner workers, Actor, Carpenter, Chimney Sweeper, Road workers
3	Walking in the woods	
3	Recr. Sports	Table tennis, Dancing, Running on even surface, Water polo, Windsurfing
3	Sports	Bodybuilding, Bowling, Curling, Cycling, Golf, Sailing, Swimming, Riding
2	Work	Sales assistent, Car repairman, Assistant, Preschool teacher, Military, Watchman, Waiter, Nurse
2	Walking on uneven surface	
2	Recr. Sports	Bodybuilding, Bowling, Curling, Cycling, Golf, Sailing, Swimming, Riding
1	Work	Buss driver, Pilot, Hairdresser, Chef, Office worker, Physician, Teacher, Secretary
1	Walking on even surface	
1	Sports	Bridge, Archery, Canoeing, Chess, Shooting

Sports	Training and competition on a regular basis
Recr. Sports	Recreational sports "for the fun of it"
