# Supplementary material for: Validation of an 18-item version of the Swedish Knee Self-Efficacy Scale for patients after ACL injury and ACL reconstruction
Source: J Exp Orthop. 2021 Oct 25;8:96. doi: 10.1186/s40634-021-00414-2 (PMC8542523; doi:10.1186/s40634-021-00414-2)

**Additional file 2**

The frequency of total score for the K-SES_18_ subscale *present* and the K-SES_18_ subscale *future* across follow-ups 10 weeks to 18 months after ACL-injury and ACL-reconstruction.


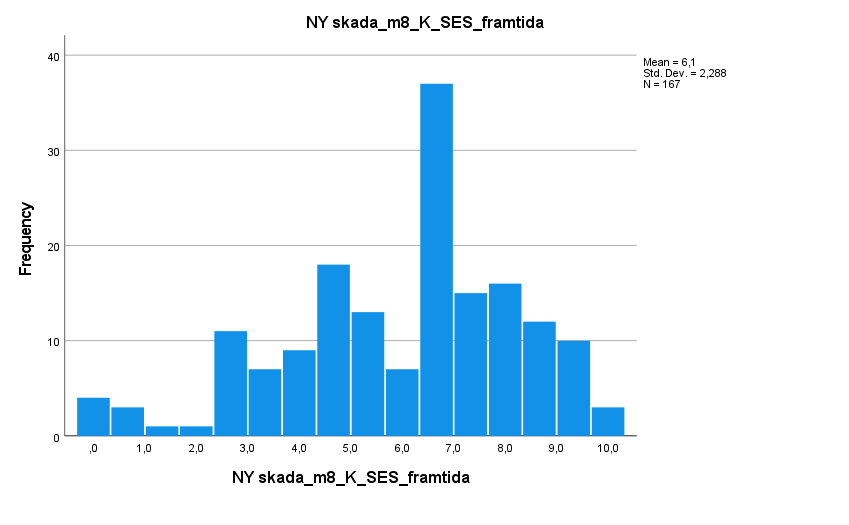

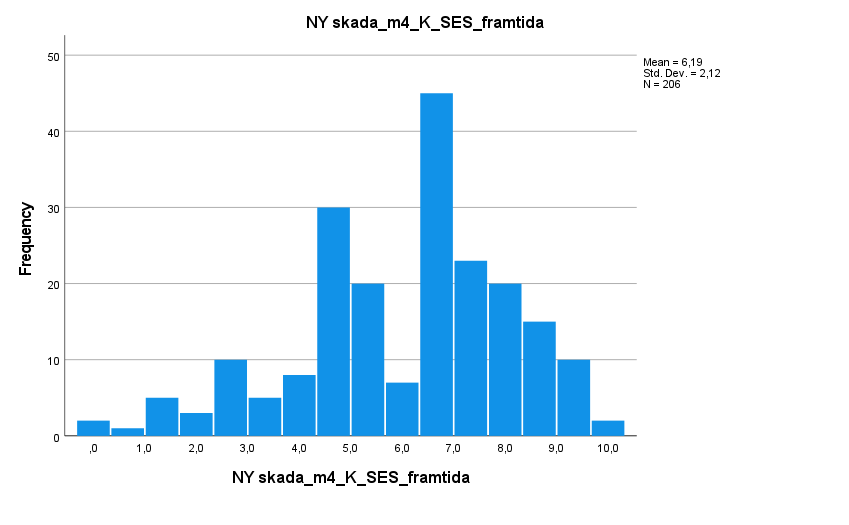

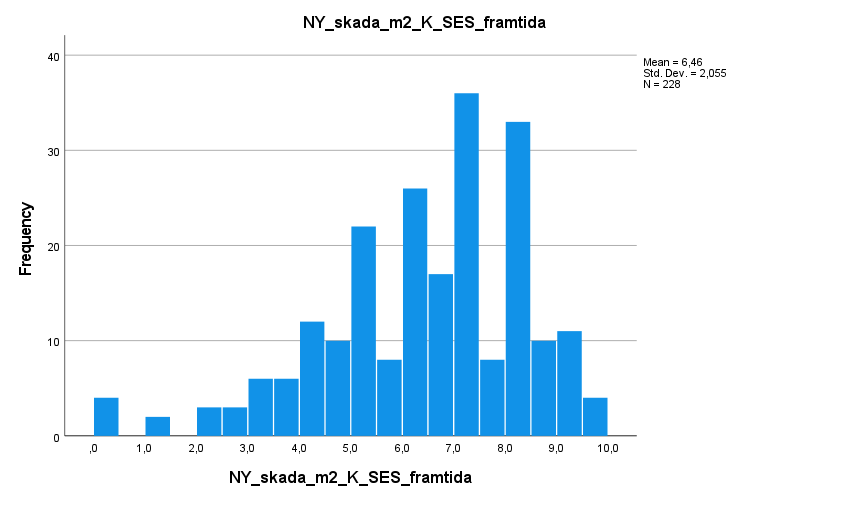

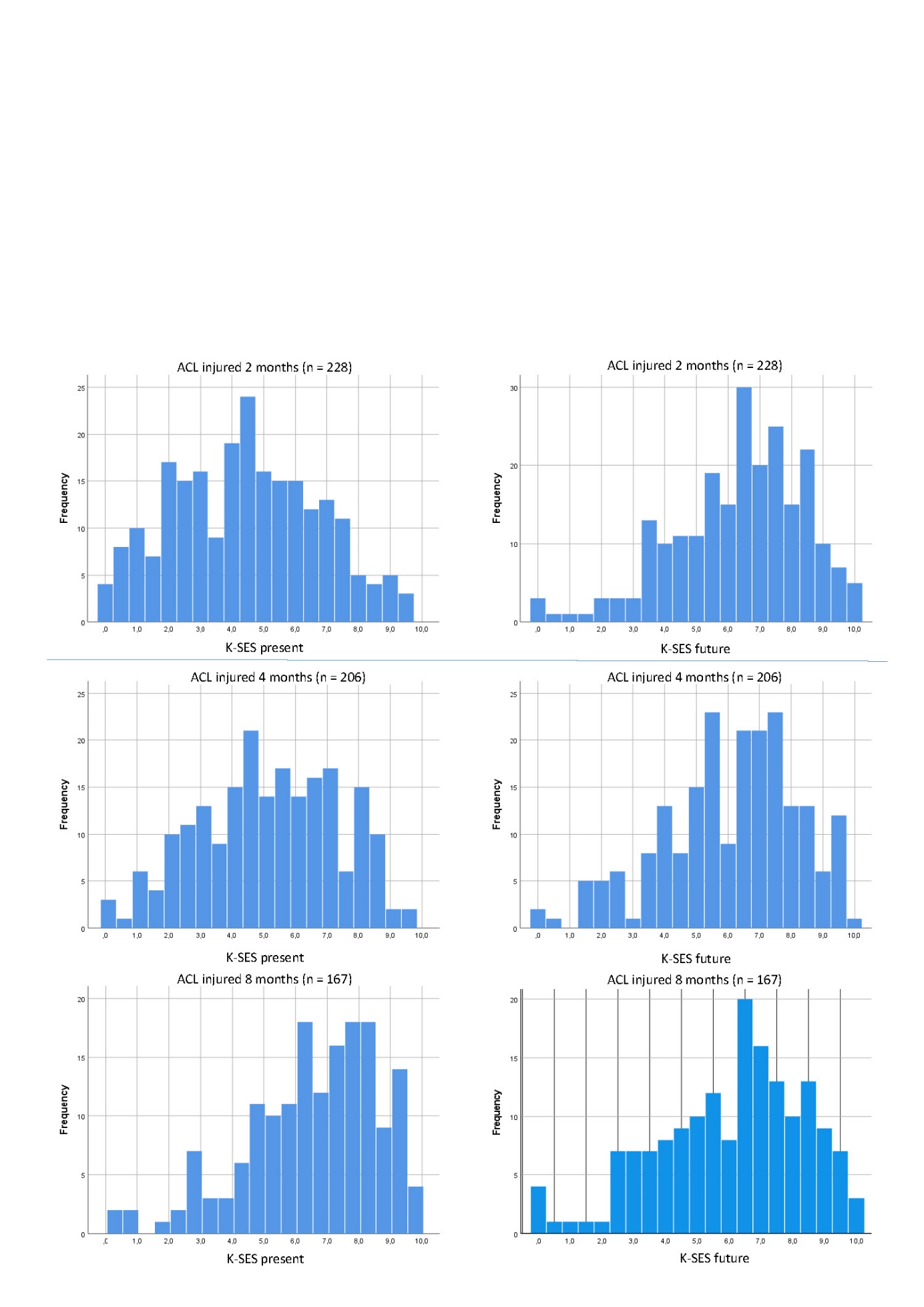


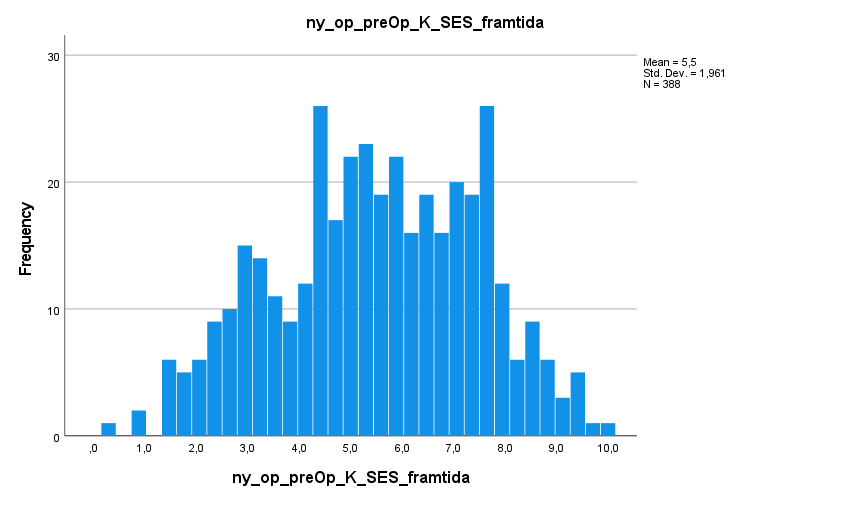

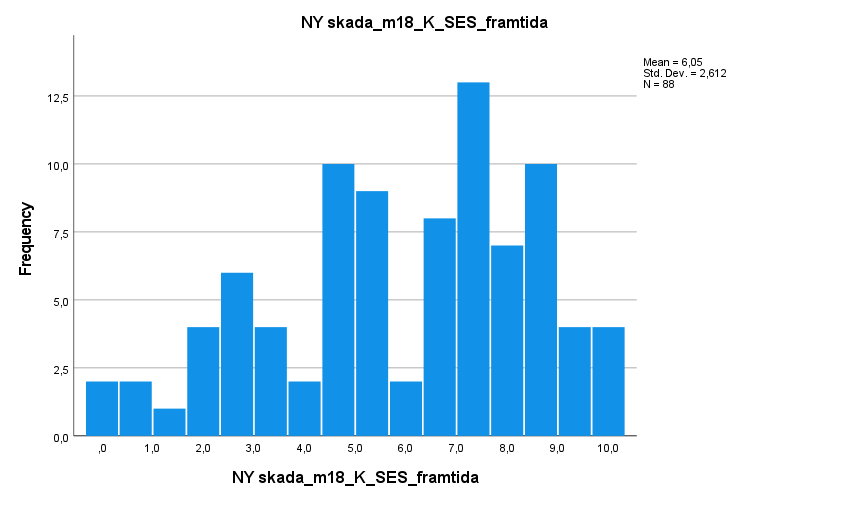

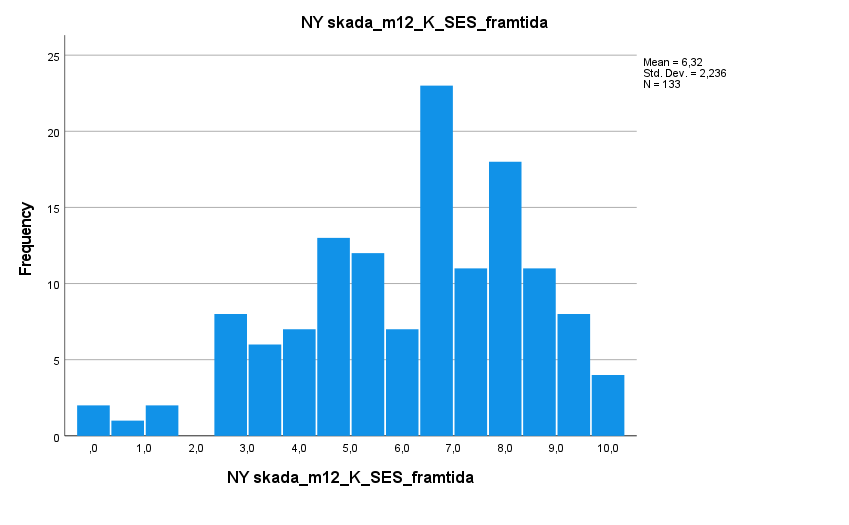

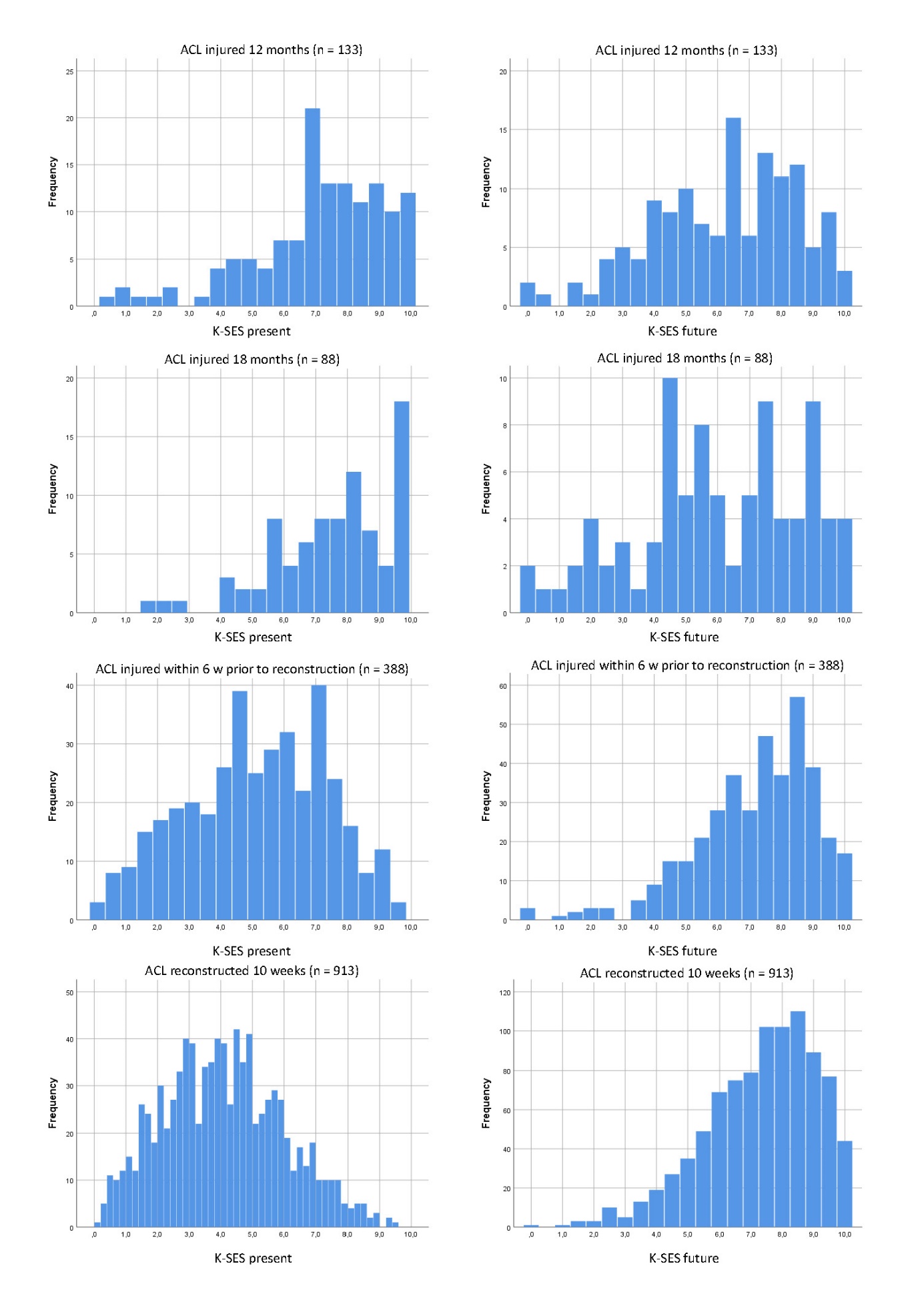


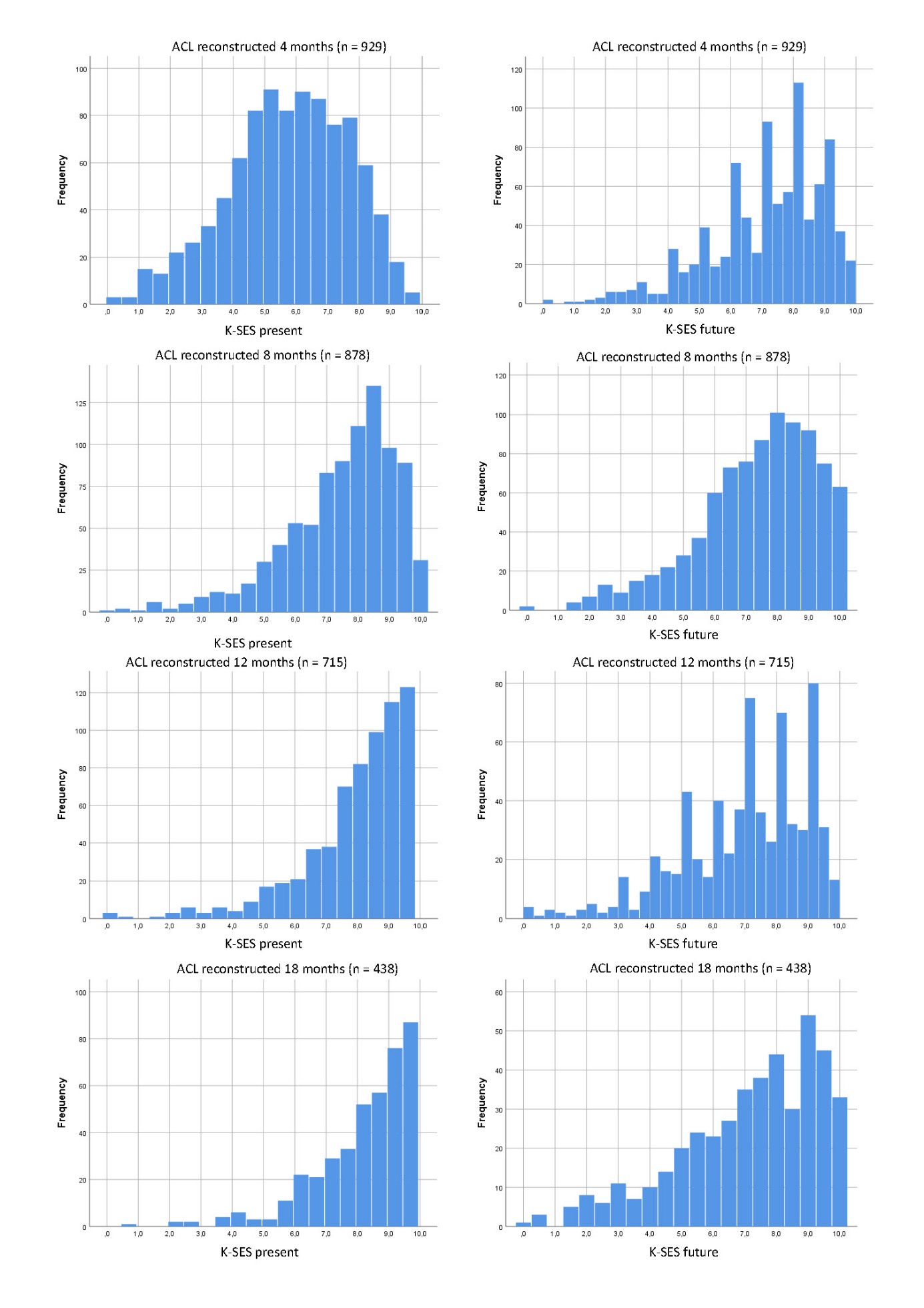

Supplement: Supplementary file 2 — Additional file 2. The frequency of total score for the K-SES18 subscale present and the K-SES18 subscale future across follow-ups 10 weeks to 18 months after ACL-injury and ACL-reconstruction. Frequency diagrams of K-SES18 subscales present and future for all 11 follow-ups as an illustration of floor and ceiling effects. [file 40634_2021_414_MOESM2_ESM.docx]
